# Evaluating Large Language Models for Diagnostic Accuracy and Health Information Quality in Oral Mucosal Diseases

**DOI:** 10.3390/jpm16030129

**Published:** 2026-02-27

**Authors:** Melisa Iacob, Ayham Qawas, Ramesh Balasubramaniam, Agnieszka M. Frydrych, Omar Kujan

**Affiliations:** 1 Toothbuds, Perth, WA 6062, Australia; melisa.iacob@outlook.com; 2 UWA Dental School, The University of Western Australia, Nedlands, WA 6009, Australia; 24188258@student.uwa.edu.au (A.Q.); ramesh.balasubramaniam@uwa.edu.au (R.B.); agnieszka.frydrych@uwa.edu.au (A.M.F.); 3 Oral Pathology Discipline, Oral and Maxillofacial Biology & Diseases Research Stream, UWA Dental School, The University of Western Australia, 17 Monash Avenue, Nedlands, WA 6009, Australia

**Keywords:** artificial intelligence, ChatGPT, oral mucosal diseases, readability, diagnostic accuracy, health information quality

## Abstract

**Background**: Multimodal large language model (MLLM)-based systems capable of generating health-related information and diagnostic suggestions are increasingly used for health information retrieval; however, their accuracy, readability, and quality in oral healthcare remain unclear. Oral mucosal diseases comprise a heterogeneous group of conditions affecting the oral lining, ranging from benign and reactive lesions to potentially malignant and malignant disorders. Objective: This study evaluated and compared the diagnostic performance, readability, and information quality of MLLMs with traditional search engines included as comparator platforms, in diagnosing oral mucosal diseases. **Methods**: A cross-sectional observational study was conducted using 100 validated oral mucosal case scenarios representing benign, malignant, potentially malignant, infectious, and reactive oral lesions. Each scenario was entered into ChatGPT 3.5, ChatGPT 4.5 (Plus), Microsoft Copilot (smart), Grok (xAI), Claude (Sonnet 4.5), DeepSeek v3.1, and search engines Google, Bing, and Yahoo. Diagnostic accuracy, Positive Predictive Value (PPV), and Negative Predictive Value (NPV) were compared against reference diagnoses. Information quality was assessed using the DISCERN tool, and readability was evaluated using Flesch–Kincaid Reading Ease (FRES) and Grade Level (FKGL) scores. Statistical analyses included Cochran’s Q and McNemar tests (*p* < 0.05). **Results**: ChatGPT 4.5 demonstrated the highest overall diagnostic accuracy (88.5%), PPV (92%), and NPV (88%), followed by DeepSeek v3.1 and Claude (Sonnet 4.5). Traditional search engines performed poorly (accuracy 18–55%). MLLMs achieved higher DISCERN scores (2.84–3.20) but lower readability (FKGL = 11–14) than search engines (FKGL = 6–7). No platform met the recommended sixth-grade reading level for consumer health information. **Conclusions**: MLLMs, particularly ChatGPT Plus (GPT-4.5), outperformed conventional search engines in diagnostic accuracy and content quality but produced complex, less-readable text. Future AI development should prioritise improving clinical accuracy alongside readability and transparency to ensure equitable access to reliable oral health information.

## 1. Introduction

Oral diseases encompass a spectrum of conditions, including orofacial pain, temporomandibular disorders, salivary gland dysfunction, oral cancer, periodontal disease, and mucosal lesions. These diseases are recognised as a global public health challenge, adversely affecting systemic health, psychological well-being, and quality of life [1]. Despite this, population-level data on the prevalence and distribution of oral mucosal diseases remain limited, with studies reporting a wide prevalence range between 5% and 65%, largely due to geographic and methodological variability [2,3]. In Australia, the estimated prevalence among adults is approximately 20.5% [4], indicating a significant burden on healthcare systems and underscoring the need for improved diagnostic strategies.

Recent analyses of global disease burden further highlight the rising incidence and mortality of lip and oral cavity cancers, especially in low-resource settings [5]. The need for diagnostic tools that are timely, precise, and widely accessible is increasingly pressing, especially because oral mucosal lesions can be subtle and are frequently missed or misinterpreted, given the anatomical complexity of the oral cavity [6].

The complexity of diagnosing oral mucosal diseases arises from the oral cavity’s intricate anatomy, the subtlety of early clinical presentations, and variability in clinician interpretation [7]. These challenges have catalysed interest in the use of artificial intelligence (AI) to support diagnostic accuracy, consistency, and accessibility. AI refers to computational systems designed to perform tasks that typically require human intelligence, including pattern recognition, reasoning, and decision-making. AI chatbots generate human-like responses to user queries, and some are powered by multimodal large language models (MLLMs) that process both text and image inputs. In this study, the term “AI chatbots” is used to refer specifically to chatbot platforms powered by MLLMs. Multimodal Large Language Models, including ChatGPT and Microsoft Copilot, have emerged as widely used tools for information dissemination in healthcare. These systems can generate human-like responses, assisting in clinical education, patient communication, and preliminary diagnostic support [8,9,10].

Notably, recent advancements in AI have expanded capabilities beyond text generation to include image recognition and multimodal input processing. Despite their growing popularity, there is a critical need to evaluate the diagnostic reliability, content quality, and readability of AI-generated health information, particularly in areas as nuanced as oral mucosal pathology. While early investigations into AI applications in oral health have demonstrated promising potential [11,12,13], most have focused on individual models in isolation, lacking comprehensive cross-platform analysis.

In parallel, conventional internet search engines such as Google, Bing, and Yahoo! are web-based information retrieval systems that index and rank content from external websites in response to user queries and continue to be primary sources of health information for the public, despite variability in content accuracy and complexity. Although studies report that patients often find this information trustworthy [14], others argue that the language level remains prohibitively complex for individuals with limited health literacy [15]. The American Medical Association recommends that health materials be written at or below a sixth-grade reading level to ensure broader accessibility [15,16].

This study aims to conduct a comparative evaluation of leading MLLMs, ChatGPT 3.5, ChatGPT Plus (GPT-4.5), Microsoft Copilot (smart), Claude (Sonnet 4.5), Grok (xAI), and DeepSeek (v3.1) against globally used search engines (Google, Bing, Yahoo!) in the context of diagnosing oral mucosal diseases. We assess these platforms based on diagnostic accuracy, information quality, and readability, offering a multidimensional analysis of their clinical utility in oral health education and self-diagnosis.

## 2. Methods

### 2.1. Study Design and Case Selection

This was a cross-sectional observational study approved by the University of Western Australia Human Research Ethics Committee (2024/ET000394). A total of 100 oral mucosal case scenarios were used, each comprising a clinical image and corresponding patient-presenting complaint. These scenarios were extracted from the electronic oral medicine records archive and represented a wide spectrum of diagnoses, including benign, malignant, and normal anatomical conditions. All cases were previously reviewed and validated by a panel of oral medicine specialists. Of these, 26 cases were utilised in prior peer-reviewed research [7]. All clinical images were obtained in the same clinical setting using a dedicated clinical camera under consistent conditions and standardized as clinically feasible with respect to lighting, framing and resolution. Any variations reflect unavoidable clinical constraints rather than intentional differences. Identical images were submitted across all platforms with no alterations. For platforms that permitted combined image and text input, contextual information was provided in accordance with each platform’s functional capabilities.

### 2.2. Search Strategy

We evaluated the diagnostic performance and information quality of three global search engines (Google, Bing, Yahoo!) and six MLLMs: ChatGPT 3.5 (initially released November 2022), ChatGPT Plus (GPT-4.5; released early 2025), Microsoft Copilot, Claude (Sonnet 4.5), Grok, and DeepSeek v3.1 with data collected between April 2024 and April 2025.

Each clinical scenario was presented to all platforms using the same prompt:

“What are the possible conditions that could cause this?”

For chatbot interactions, where image input was supported (ChatGPT 3.5, Grok, Claude, DeepSeek v3.1, ChatGPT Plus (GPT-4.5)), Copilot), both the image and chief complaint were submitted.

To avoid memory bias and reinforcement, each case was posed in a new chat window for all AI tools. Search engine results were limited to the first 20 websites returned. No geographic or language filters were applied at the search stage to reflect real-world user behaviour; however, retrieved results were subsequently screened for eligibility. Inclusion criteria comprised text-based, English-language content that provided diagnostic or explanatory information relevant to the presented oral mucosal condition and appeared within the first 20 search results for each query.

Exclusion criteria included:

- Irrelevant content;
- Non-health-related blogs;
- Videos;
- Broken or paywalled links;
- Non-English content.

Results from all platforms were compiled and stored on an external hard drive for evaluation.

### 2.3. Diagnostic Accuracy Assessment

Suggested diagnoses from each platform were evaluated against the definitive diagnosis from the validated clinical case. A diagnosis was classified as correct if it either:

1. Directly matched the confirmed diagnosis;
2. Had the correct diagnosis within the top three differential diagnoses.

The assessment was conducted on two separate occasions for the same set of oral lesions, with both rounds completed by the same researcher to evaluate intra-rater reliability. This approach was adopted given the well-recognised risk of hallucinations in chatbot-generated outputs. Where discrepancies occurred between rounds, the first response was used for analysis.

### 2.4. Information Quality Assessment

The DISCERN instrument was used to evaluate the quality of health-related written information [17,18]. This validated tool includes 16 questions divided into:

- Section 1: Reliability (Q1–8);
- Section 2: Quality of treatment information (Q9–15);
- Section 3: Overall rating (Q16).

Each question was rated on a 5-point Likert scale, where 1 = not fulfilled, 5 = fully met. For MLLMs responses, Questions 4 and 5 (source attribution and publication date) were omitted, as these platforms do not provide references.

Two independent assessors (M.I. and O.K.) rated the responses. Prior to formal scoring, both assessors followed a standardised scoring protocol based on the DISCERN guidelines to ensure consistent interpretation of each item. Discrepancies were resolved by consensus after blinded re-evaluation [18].

### 2.5. Readability Assessment

Text excerpts (200–500 words) from each search engine/webpage and MLLMs were analysed using the Flesch Reading Ease Score (FRES) and Flesch–Kincaid Reading Grade Level (FKRGL), via www.readabilityformulas.com; excerpts consisted of the first 200–500 words of each output, with shorter responses analysed in full, and were selected sequentially rather than randomly. These tools estimate reading complexity based on average sentence length and syllables per word [18].

Scoring formulas:

- FRES = 206.835 − (1.015 × ASL) − (84.6 × ASW);
- FKRGL = (0.39 × ASL) + (11.8 × ASW) − 15.59.

where ASL = average sentence length, and ASW = average syllables per word.

FRES interpretations:

- 90–100 = very easy;
- 80–89 = easy;
- 70–79 = fairly easy;
- 60–69 = standard;
- 50–59 = fairly difficult;
- 30–49 = difficult;
- 0–29 = very difficult.

FKRGL scores were used to approximate the educational level required to comprehend the content (e.g., grade 6 = age~12).

### 2.6. Statistical Analysis

Statistical analysis was performed to evaluate the quality and readability outcomes across platforms. Differences in non-parametric variables, including quality and readability scores, were assessed using the Mann–Whitney U test. To examine associations between quality (as measured by the DISCERN tool) and readability metrics (Flesch–Kincaid Grade Level and Flesch Reading Ease), chi-square tests were employed. To account for multiple comparisons and reduce the likelihood of Type I error, the Benjamini–Hochberg procedure was applied to adjust the false discovery rate. Inter-rater reliability of the chatbot was assessed using Cohen’s Kappa test. The diagnostic accuracy of the tested tool was determined by calculating the proportion of answers scored as correct relative to the total number of answers generated, together with its 95% confidence interval, using Wald’s binomial method. A *p*-value of less than 0.05 was considered indicative of statistical significance throughout the analyses. All statistical analyses were conducted using IBM SPSS Statistics for Windows, Version 29.0 (IBM Corp., Armonk, NY, USA).

## 3. Results

### 3.1. Readability and Quality of Information

As shown in Table 1, the quality of information varied substantially across MLLMs and search engines. Among all platforms, ChatGPT 4.5 (Plus) achieved the highest DISCERN score (mean = 3.20), indicating a “good” quality level, followed by Microsoft Copilot (2.96) and DeepSeek v3.1 (2.99). In contrast, conventional search engines (Google, Bing, Yahoo) scored poorly, with mean DISCERN ratings below 1.7, representing “very poor” reliability and treatment information quality.

Readability, measured using the FRES and FKGL, revealed that AI-generated content was significantly more complex than that retrieved from search engines. ChatGPT Plus (GPT-4.5) demonstrated the lowest readability (FRES = 41.04, FKGL = 14.05), corresponding to a professional-level reading grade, with 37% of responses classified at a college-level and 8% at a professional level. In contrast, Bing and Yahoo presented easier-to-read text (FRES ≈ 67; FKGL ≈ 6.8), but these outputs lacked depth and clinical relevance. Claude (Sonnet 4.5) and Grok provided moderately readable responses (FRES = 52.15 and 56.42, respectively), indicating a balance between accessibility and content depth.

Overall, readability scores demonstrated an inverse relationship with quality; higher-quality, clinically focused AI responses required higher reading proficiency.

### 3.2. Diagnostic Accuracy

**Figure 1:**
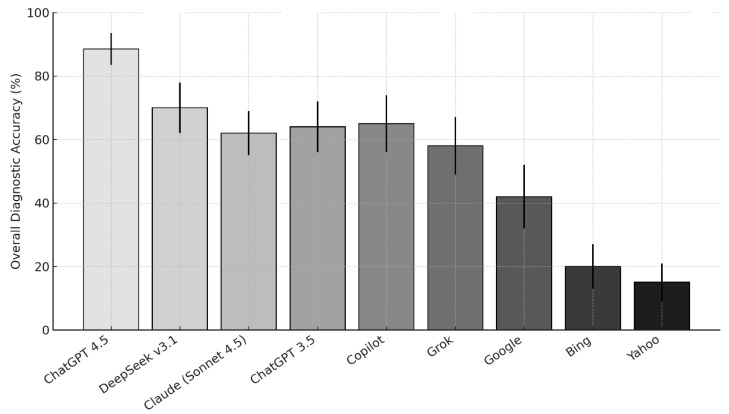
Diagnostic accuracy of MLLMs and search engines for oral mucosal diseases. The figure shows the proportion of correct diagnoses generated by different MLLMs and search engines using identical oral mucosal cases.

Figure 1 and Table 2 present the diagnostic accuracy of MLLMs and search engines across oral mucosal disease categories. Accuracy varied significantly between platforms (Cochran’s Q *p* < 0.001 across all categories).

ChatGPT Plus (GPT-4.5) consistently outperformed all other tools across every diagnostic group, achieving an overall diagnostic accuracy of 88.5%. It performed best in identifying oral potentially malignant disorders (94%), followed by benign (90%) and oral infectious lesions (89%).

DeepSeek v3.1 achieved the second-highest performance (overall ~74–75%), particularly for benign and OPMD cases. Claude (Sonnet 4.5) and ChatGPT 3.5 demonstrated moderate diagnostic abilities (60–73%), while Copilot and Grok yielded similar but slightly lower accuracies (57–67%). Traditional search engines (Google, Bing, Yahoo) performed substantially worse, with Bing (35%) and Yahoo (25%) showing the lowest diagnostic accuracy overall.

The differences were statistically significant across lesion types (e.g., OPMDs: Q = 67.889; benign = 81.356; infections = 42.462; all *p* < 0.001).

Interestingly, intra-rater reliability for correct/incorrect classification was highest for ChatGPT Plus (GPT-4.5) (agreement ≈94%; κ ≈ 0.73), followed by DeepSeek v3.1 (≈88%; κ ≈ 0.69) and Claude (Sonnet 4.5) (≈85%; κ ≈ 0.65), and was lower for ChatGPT 3.5 (≈83%; κ ≈ 0.63), Copilot (≈80%; κ ≈ 0.58), and Grok (≈78%; κ ≈ 0.55); exact-diagnosis stability showed a similar pattern (agreement ≈68–86%; κ ≈ 0.62–0.84), indicating generally substantial repeatability for the higher-performing MLLMs and moderate repeatability for the remaining tools.

### 3.3. Predictive Value Analysis

**Figure 2:**
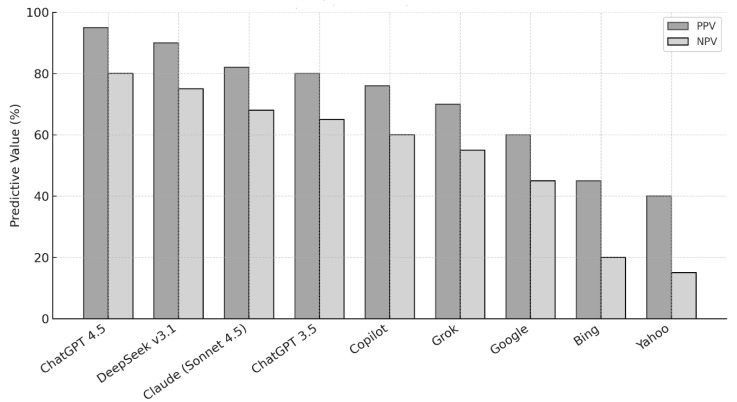
Predictive values (PPV and NPV) of MLLMs and search engines in diagnosing oral mucosal diseases. The figure shows the positive and negative predictive values of different MLLMs and search engines when evaluated using identical oral mucosal disease cases.

As illustrated in Figure 2 and Table 3, the Positive Predictive Value (PPV) and Negative Predictive Value (NPV) patterns mirrored diagnostic accuracy trends.

ChatGPT Plus (GPT-4.5) achieved the highest mean PPV (92%) and NPV (88%) across all lesion categories, indicating strong confidence in both positive and negative diagnostic predictions. The NPV remained particularly high for malignant lesions (92%), suggesting reliable exclusion of serious disease when predicted negatively.

DeepSeek v3.1 and Claude (Sonnet 4.5) followed, showing balanced PPV/NPV performance (PPV = 65–75%; NPV = 70–82%). ChatGPT 3.5 and Copilot maintained moderate predictive values, while Grok achieved slightly better NPVs (up to 80%) but lower PPVs (60–65%). In contrast, search engines performed poorly, with Google (PPV ≈ 50%), Bing (PPV ≈ 25%), and Yahoo (PPV ≈ 12%), indicating a high frequency of false-positive and false-negative diagnostic outputs.

These findings highlight that ChatGPT Plus (GPT-4.5) produced the most consistent and comprehensive diagnostic suggestions among the evaluated AI tools and search engines across the assessed metrics.

## 4. Discussion

This study represents one of the first comprehensive comparisons of MLLMs and traditional search engines for diagnosing oral mucosal diseases, integrating diagnostic performance with information quality and readability. Overall, MLLMs, particularly ChatGPT Plus (GPT-4.5), consistently outperformed conventional search engines in diagnostic accuracy and content quality. However, this performance advantage was tempered by suboptimal readability, with most AI-generated responses written above the recommended sixth-grade level for consumer health information [15,19].

Across all lesion categories, ChatGPT Plus (GPT-4.5) achieved the highest diagnostic accuracy (88.5%) and predictive performance (PPV 92%, NPV 88%). This is consistent with the emerging literature suggesting that newer-generation MLLMs can support structured clinical reasoning and differential diagnosis in complex medical contexts [20,21,22]. In contrast, search engines (Google, Bing, Yahoo) showed substantially lower diagnostic accuracy (18–55%), reinforcing prior work that highlights their limited capacity to provide coherent, case-specific diagnostic guidance without substantial user expertise in filtering and synthesis [14,23]. Notably, higher-performing MLLMs were also more internally consistent, demonstrating greater repeatability across repeated prompting of the same cases, whereas lower-performing tools showed greater output variability—likely reflecting the probabilistic nature of generative systems and sensitivity to contextual factors. Importantly, even the best-performing model exhibited occasional within-tool inconsistency, underscoring the importance of transparent reporting of repeat-testing procedures and prespecified decision rules for discrepant outputs.

The substantial performance gap between MLLMs and search engines underscores the growing capacity of generative AI to interpret multimodal data, including clinical images and symptom descriptions, demonstrating performance comparable to experienced clinicians [7]. This reflects the broader trend in healthcare AI, where large language models are increasingly demonstrating capabilities comparable to those of medical professionals in structured diagnostic reasoning tasks [8,9]. Furthermore, our findings suggest that DeepSeek v3.1 and Claude (Sonnet 4.5) represent strong emerging competitors, achieving diagnostic accuracy comparable to that of ChatGPT 3.5 while maintaining moderate readability and reliability scores.

While ChatGPT Plus (GPT-4.5) demonstrated the highest overall accuracy, its diagnostic outputs sometimes contained hallucinations, a known limitation where generative models fabricate details inconsistent with medical facts [24]. Such inaccuracies pose risks when used unsupervised by non-clinical users, highlighting the need for integration of evidence-based medical databases and structured validation pipelines to enhance reliability [25,26].

The DISCERN assessment revealed that MLLMs generally produced higher-quality content (mean DISCERN = 2.84–3.20) than search engines (1.42–1.61). However, none achieved a perfect score of 5, indicating that even the most advanced AI tools often failed to provide comprehensive or balanced treatment discussions. Similar limitations have been noted in studies evaluating ChatGPT-generated cancer and oral health information [9,22], where AI responses were frequently missing source citations and evidence hierarchies.

Readability analysis confirmed that AI-generated outputs are written well above the recommended sixth-grade level, often requiring college-level literacy (FKGL = 11–14). These findings mirror recent studies showing that even when prompted to “simplify” their responses, ChatGPT and similar models rarely achieve layperson readability [27,28]. This gap in accessibility limits the usefulness of these tools for the general public and highlights the need for plain-language adaptation frameworks and user-centred AI design [19].

In contrast, search engines such as Bing and Yahoo provided text closer to the recommended readability range (FKGL ≈ 6–7) but with limited clinical value and outdated or fragmented content. These findings reinforce a consistent pattern in the literature where higher-quality medical content tends to be less readable [14,23].

### 4.1. Implications for Clinical and Consumer Use

The strong performance of multimodal AI tools such as ChatGPT Plus (GPT-4.5) and Copilot suggests potential for integration into clinical education, triage, and preliminary patient counselling. Beyond diagnostic applications, emerging evidence suggests that AI platforms may support broader decision support functions, including the assessment of dental drug–drug interactions [24]. However, ethical considerations remain paramount. AI hallucinations and lack of source transparency can propagate misinformation, increase patient anxiety, and undermine healthcare trust [24]. For safe implementation, AI outputs should be viewed as decision support tools, not replacements for clinical expertise.

In public health contexts, chatbots can improve access to oral healthcare information, especially for underserved populations, including those in rural or resource-limited settings [29]. When appropriately monitored, AI systems may enhance disease awareness, encourage preventive care, and support early detection, potentially reducing diagnostic delays. Future systems should incorporate dynamic readability optimisation, adaptive questioning, and cross-language support to improve equity in access to health information.

### 4.2. Limitations and Future Research

Key strengths include the use of a large, clinically validated set of 100 oral mucosal case scenarios spanning benign, malignant, potentially malignant, and reactive conditions, reviewed by oral medicine specialists. The evaluation used a standardised, reproducible framework with consistent prompts (and images where applicable), and predefined criteria for diagnostic accuracy, information quality, and readability across all platforms.

Limitations include the exploratory design and an English-only evaluation, which may constrain generalisability to culturally and linguistically diverse settings. Real-world patient querying also differs from standardised vignettes due to variability in health literacy, colloquial/culturally specific symptom descriptions, incomplete histories, and multi-turn interactions that can influence outputs and safety advice. In addition, defining “correct diagnosis” as inclusion within the top three differentials may overestimate clinical utility, particularly for malignant and potentially malignant disorders where the top-ranked diagnosis and escalation guidance are most consequential. Finally, rapid MLLM updates may reduce reproducibility despite identical methods. Future studies should use multilingual/culturally diverse datasets, test multi-turn conversations, report stricter rank-based metrics (e.g., top-1 accuracy or weighted scoring), and prospectively evaluate longitudinal reasoning and safety behaviours (including escalation advice) with independent validation and bias auditing [30].

## 5. Conclusions

MLLMs substantially outperformed traditional search engines in diagnostic accuracy, information quality, and predictive value for oral mucosal diseases, with ChatGPT Plus (GPT-4.5) achieving the strongest overall performance, followed by DeepSeek v3.1 and Claude (Sonnet 4.5). Nonetheless, readability remains a major limitation, and hallucinations and inconsistency, although reduced in higher-performing models, support the need for cautious clinical interpretation. Integrating MLLMs with evidence-based sources, expert oversight, and dynamic readability optimisation will be essential for safe and equitable deployment in oral healthcare.

## Figures and Tables

**Table 1:** DISCERN, Flesch–Kincaid Reading Ease (FRES), and Flesch–Kincaid Grade Level (FKGL) scores from MLLMs and search engines regarding oral mucosal diseases.

Platform	DISCERN (Q16)	DISCERN IQRange	FRES	FRES IQRange	FKGL	FKGL Range	College-Level (%)	Professional Level (%)
**Bing**	1.46	1.75–1.00	67.63	63.00–73.00	6.80	5.67–7.31	0	0
**Yahoo**	1.42	1.00–1.00	67.63	60.00–75.50	6.89	5.77–7.61	0	0
**Google**	1.61	2.00–1.00	64.33	59.00–69.00	7.57	6.40–8.46	2	0
**ChatGPT 3.5**	2.73	3.00–2.25	43.58	38.00–49.75	12.31	11.27–13.35	26	4
**ChatGPT 4.5 (Plus)**	3.2	3.00–3.50	41.04	38.50–48.75	14.05	11.85–14.11	37	8
**Microsoft Copilot**	2.96	3.00–3.00	44.08	37.25–50.75	10.67	9.99–11.71	7	1
**Claude (Sonnet 4.5)**	2.92	2.95–3.12	52.15	36.00–64.44	11.10	10.66–12.34	9	3
**Grok**	2.84	2.86–3.04	56.42	42.00–62.10	7.88	6.84–10.68	7	2
**DeepSeek v3.1**	2.99	2.92–3.00	54.25	36.64–66.40	8.68	7.42–10.80	8	4

**Table 2:** Diagnostic Accuracy (%) by Lesion Category with Statistical Significance.

Platform	Benign Oral Lesions	Malignant Lesions	OPMDs	Oral Infections	Reactive Oral Lesions
**ChatGPT 4.5**	90 *	85 *	94 *	89 *	87 *
**DeepSeek v3.1**	74	68	75	73	69
**Claude (Sonnet 4.5)**	72	66	72	66	63
**ChatGPT 3.5**	70	63	73	68	65
**Copilot**	65	60	67	64	61
**Grok**	61	56	65	60	57
**Google**	55	50	58	53	50
**Bing**	35	28	40	33	30
**Yahoo**	25	18	28	22	20

Note: The * symbol indicates the best-performing tool in the category. Cochran’s Q test results by category: OPMDs: Q = 67.889, *p* < 0.001; Oral infections: Q = 42.462, *p* < 0.001; Benign oral lesion: Q = 81.356, *p* < 0.001; Reactive oral lesion: Q = 55.96, *p* < 0.001; Malignant lesions: Q = 42.2, *p* < 0.001.

**Table 3:** Positive and Negative Predictive Values (PPV and NPV) by Lesion Category Across MLLMs and Search Engines.

Platform	Benign Oral Lesions (PPV/NPV)	Malignant Lesions (PPV/NPV)	OPMDs (PPV/NPV)	Oral Infections (PPV/NPV)	Reactive Oral Lesions (PPV/NPV)
**ChatGPT 4.5**	92/88	70/92	85/90	80/85	91/92
**DeepSeek v3.1**	65/50	60/80	72/76	75/82	72/70
**Claude (Sonnet 4.5)**	60/45	55/78	68/74	70/80	66/64
**ChatGPT 3.5**	55/30	30/75	55/68	60/73	60/58
**Copilot**	60/35	45/80	65/70	68/77	55/53
**Grok**	55/30	50/85	60/75	60/80	60/60
**Google**	50/25	45/80	60/70	65/75	50/45
**Bing**	25/15	20/50	30/40	25/45	28/25
**Yahoo**	12/8	8/15	15/20	10/18	12/10

PPV (Positive Predictive Value), NPV (Negative Predictive Value).

## Data Availability

The raw data supporting the conclusions of this article will be made available by the authors on request.
